# The Influence of Functional Movement and Strength upon Linear and Change of Direction Speed in Male and Female Basketball Players

**DOI:** 10.5114/jhk/177313

**Published:** 2024-04-25

**Authors:** Francisco J. Barrera-Domínguez, Bartolomé J. Almagro, Jorge Molina-López

**Affiliations:** 1 Faculty of Education, Psychology and Sport Sciences, COIDESO, University of Huelva, Huelva, Spain.

**Keywords:** multidirectional, agility, sprint, performance, team sport, gender

## Abstract

The present study aimed to analyse the relationship between functional movement and strength variables upon linear speed (Ls) and change of direction (COD) based on gender. It also aimed to identify the determinants of performance of Ls and COD according to gender. Fifty basketball players (54% female) completed the assessment in which the weight-bearing dorsiflexion test, the Y-balance test, the unilateral countermovement jump, the unilateral drop jump, the unilateral triple hop test, Ls and CODs were performed. Speed variables were divided according to time execution into “low-performance” and “high-performance” to establish a comparison between performance groups. Strength variables significantly influenced speed tests’ performance in both genders (p < 0.05). For males, the greater the Ls, the higher the change of direction deficit (p < 0.001). Multiple regression analysis revealed that a long and vertical stretch-shortening cycle (SSC) was the most influential physical ability for speed performance in females (45–65% variance explained; p < 0.001), while in males, a short and horizontal SSC played a significant role (30–61% variance explained; p < 0.022). These results suggest that gender should be considered in programming strength training to improve speed, as each gender will benefit most from the application of different force-orientations and different SSC. Also, the faster the male players were in Ls, the less efficient they were in the COD performance. This is why for men, it would be recommended to perform eccentric exercises along with deceleration and technique drills to improve COD speed.

## Introduction

Change of direction (COD) actions are most frequent during a basketball game regardless of the playing position (Salazar et al., 2020). COD is defined as the ability to decelerate and accelerate towards a different direction in a planned manner (Nimphius et al., 2018). Although, during real game, directional changes occur mainly in reaction to external stimuli (e.g., movement of the ball or an opponent) and these agility actions may not have a direct relationship with COD performance, the planned COD movements represent the mechanical and physiological basis for understanding the real situation. In basketball, 15.1% of these actions are performed at maximum intensity (deceleration > –3.5 m•s^−2^) and could be decisive in the final result of the match (Svilar et al., 2018), since these actions can lead to a basket or a major change in the development of the game (Nimphius et al., 2018). COD actions are often preceded by different movements in basketball, such as linear speed in offense or lateral shuffling in a defensive context. For both the offensive and defensive purposes, these high-speed movements are essential given the short duration of decisive actions in the game. For this reason, the assessment and analysis of COD is essential to understand the needs of basketball players, being a topic of current interest to both researchers and coaches.

Previous research, which focused on studying the main determinants of COD performance (Barrera-Domínguez et al., 2020; Hewit et al., 2013; Pereira et al., 2018), has indicated that COD is a complex skill that is influenced by technique and different physical capacities such as dynamic balance, linear speed (Ls), as well as strength and power of lower limb muscles. However, there is still no consensus on which of these variables is the most relevant in the performance of COD actions and whether gender influences these actions. This might be explained by the type of a test used to assess COD speed (e.g., T-test, V-cut or 505-test) which could change the explanatory variables of this skill (Lazić et al., 2022; Pereira et al., 2018). Since there is no reference method to assess COD, each investigation usually uses a different COD test, thus the results among studies may not be comparable. Hence, the selection of a correct assessment is a critical factor when trying to assess these actions. In this sense, the biomechanical demands of COD are angle and task-dependent (Dos’Santos et al., 2018). Therefore, it would be advisable to assess this skill with several angulations to create a “COD angle profile” for each athlete (Dos’Santos et al., 2018; Gonzalo-Skok et al., 2023), rather than with a single test. Furthermore, it is interesting that many studies confirm that Ls is a determinant skill to improve COD speed performance with moderate to high correlations, depending on the type of a COD test used (Barrera-Domínguez et al., 2020; Chaouachi et al., 2009). Specifically, in young basketball players of both genders (Michael et al., 2021), it was observed that Ls explained significantly the variance of different COD tests. This could be because more than 70% of the total time of these COD tests are carried out through the Ls (Nimphius et al., 2016, 2018). In order to avoid this limitation, previous research has used a novel concept to assess COD actions in isolation from Ls, called the change of direction deficit (CODD) (Freitas et al., 2021b). The CODD indicates what percentage of the total time of the COD test has been spent exclusively on the COD action, thus isolating it from Ls.

Regarding the other physical abilities that were found to affect COD performance, research has shown that for the male gender there was a greater relationship between dynamic balance and COD performance (Gonzalo-Skok et al., 2015), with no such a relationship observed in the female gender (Sekulic et al., 2013). Previous research (Falch et al., 2020) analysing the relationship among lower limb muscle strength, power and COD speed performance, suggested that actions involving a stretch-shortening cycle (SSC), such as plyometric actions, were the most specific determinants of COD speed. However, these studies were carried out on a male sample; when female players were analysed, the plyometrics did not turn out to be as specific as maximum strength (Falch et al., 2021; Mikołajec et al., 2017; Spiteri et al., 2014). This could be due to a lower representation of female players in the scientific literature or due to usually lower levels of baseline strength in females. As it can be observed, previous scientific evidence does not reach a clear consensus about the most determinant variables of COD performance in basketball players for each gender. Moreover, this relationship between the different variables and the performance in speed actions differs considerably depending on the gender and the speed test used (Lazić et al., 2022). Therefore, more research comparing between gender differences using the same COD assessment tests is needed.

To the best of the authors’ knowledge, there is still very little research that indicates which are the most determinant factors in COD performance differentiating and comparing directly between genders, since female athletes are less represented in COD research, which contrasts with their increasing presence in competitive sport. Thus, more data would be needed on this topic. Therefore, the main aim of this study was to analyse the relationship between functional movement and strength variables in several Ls and COD speed tests based on gender. In addition, to complement this, the study aimed to identify the determinants of performance of Ls and COD according to gender. The speed tests analysed require a greater application of force in a horizontal plane (McBurnie and Dos’Santos, 2022), thus it was hypothesised that the tests evaluated which would have a greater emphasis on this direction, would be the most determinant of speed performance in both genders. It was also hypothesised that males might benefit more from a short SSC than their female counterparts, since stronger athletes benefit to a higher extent from the short SSC than weaker ones (Bourgeois et al., 2017).

## Methods

### Participants

A total of fifty basketball players, twenty-three male (age: 23.9 ± 5.70 years, body height: 187.2 ± 7.59 cm; body mass: 85.9 ± 17.1 kg) and twenty-seven female (age: 22.7 ± 3.81 years, body height: 170.5 ± 7.69 cm; body mass: 64.4 ± 7.88 kg), volunteered to participate in the study. All players had their regular basketball training at least three days a week for two hours and played one federated game per week during the season. In addition, they belonged to the same competitive level, played in the Spanish N1 National League and had at least 10 years of experience playing basketball. None of the athletes included in the study had suffered an injury in the six months prior to the study. All of them were previously informed of the possible risks and benefits of participating in the study and gave their written consent before the start of testing. This research was approved by the Andalusian Biomedical Research Ethics Committee (reference number: FBD_UHU2020; approval date: 08 October 2020) in accordance with the guidelines established in the Declaration of Helsinki.

### Design and Procedures

A cross-sectional experimental design was used to determine the association of functional movement and vertical and horizontal strength upon speed actions according to gender. Data collection was carried out during the competitive phase of the season. All participants performed two testing sessions with a time interval of 48 h in between. All assessments were undertaken just before each training session (between 19:00 and 21:00) and under the same conditions to avoid possible influences from the training track or weather. A familiarization protocol with the proposed tests was carried out in the week preceding testing. Before the start of the evaluation sessions, a 10-min warm-up was conducted, which included jogging, series of dynamic stretching and several acceleration runs, followed by specific potentiation exercises. Moreover, each player was instructed to attend the testing sessions adequately hydrated and rested, with no high intensity training in the previous 24 h, and to control their caffeine and food intake for at least 3 h before each evaluation.

The first testing session was devoted to quantitative movement tests: a weight-bearing dorsiflexion test (WB-DF) and the Y-Balance Test (YBT); and strength tests predominantly in a vertical direction: a unilateral Countermovement Jump (CMJu) and a unilateral Drop Jump (DJu). The second session included all horizontal tests: 10-m Ls and COD at different angles (45°, 90° and 180°); and the unilateral Triple Hop Test (THTu) to assess reactive strength predominantly in a horizontal direction. All players performed a total of three attempts of each test with a 2-min rest interval in between, achieving an Intraclass Correlation Coefficient (ICC) between 0.86 and 0.98, and a coefficient of variation (CV) of less than 5%. The mean of all attempts for each test was used for further analysis.

### Weight-Bearing Dorsiflexion Test (WB-DT)

Ankle dorsiflexion was assessed with My ROM App (Apple Inc., Cupertino, CA, USA) (Balsalobre-Fernández et al., 2019) by placing the mobile device on the anterior tibial crest, just below the tibial tuberosity, recording the result in degrees. Each player placed their hands on their hips, as well as the foot to be measured in front and the opposite foot resting just behind. In this position, participants were instructed to lunge forward until their knee reached the maximum range of movement. The heel was required to remain in contact with the floor at all times. Players remained barefoot for the measurement.

### Y-Balance Test (YBT)

Dynamic balance was assessed by the Y-Balance Test using the OctoBalance device (OctoBalance, Check your Motion, Albacete, Spain). Once maintaining a balanced position on one foot on the platform, each player had to reach the maximum possible distance in three directions: anterior, posteromedial and posterolateral (Onofrei et al., 2019). All attempts were supervised by the researchers and were considered valid if 1) the heel remained on the back edge of the platform and the second metatarsal was on the front line, 2) the hands were kept on the hips, and 3) the reaching foot only rested on the platform. For the final analysis, the average of the three trials was calculated and each participant's result was normalised using leg length.

### Unilateral Countermovement Jump (CMJu) and Unilateral Drop Jump (DJu)

Jump height in CMJu and DJu tests was determined using a Chronojump contact platform (Chronojump BoscoSystem®, Barcelona, Spain) (De Blas et al., 2012). Before testing, participants started with an initial position with one foot on the platform for the CMJu and on a 25-cm platform for the DJu, whereby each athlete landed with one foot on the platform with the intention of jumping as high as possible. The jump was considered valid if 1) the hands were not separated from the hips at any time, 2) the knees were not bent during the flight time, and 3) the athlete landed with only one foot on the same point from which he/she jumped, holding the position for at least 2 s. In addition, the DJu was used to calculate the reactive strength index (RSI) of each leg using the flight time/contact time ratio for each jump.

### Linear Speed (Ls) and Change of Direction (COD) Speed

**Figure 1:**
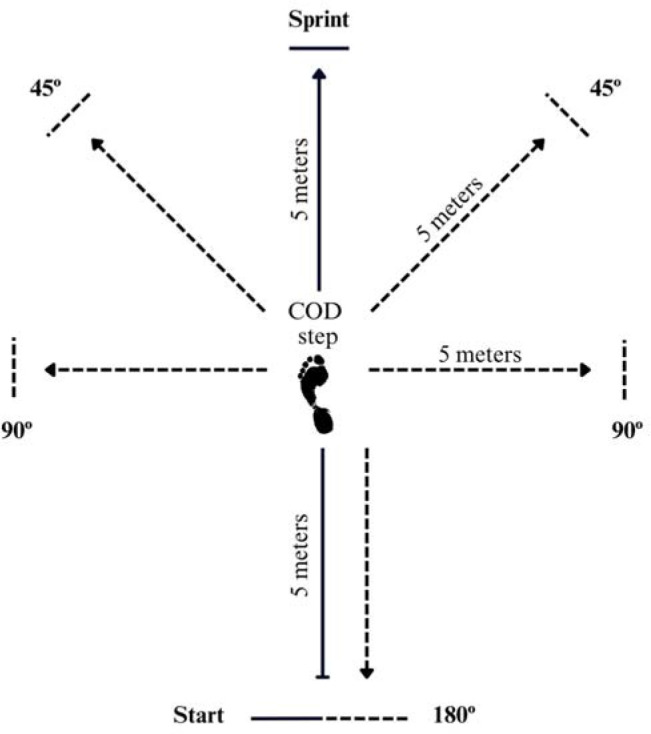
Schematic presentation of the linear speed test (10-m sprint) and the change of direction speed test (5+5 m at a 45º, 90º and 180º cut).

Execution time for speed tests was measured by Chronojump single beam photocells (Chronojump BoscoSystem^®^, Barcelona, Spain). The photocells were placed 2 m from each other with a height of 1.10 m (approximately the height of the players' hips). Before the start of the test, each player was positioned 0.5 m behind the first gate, in a two-point split stance (i.e., standing position with the preferred foot forward placed exactly 0.5 m behind the starting line). Then, each player accelerated at maximum speed to the second gate located 10 m away for all the tests, in a straight line for the linear speed test and with a turning point at 5 m where each athlete performed a COD45º, COD90º and COD180º to reach the second gate in the shortest possible time (Figure 1). CODs at 45°, 90° and 180° were performed on both sides and laterality was defined by the leg on which participants set on the court when performing the COD mechanics (Cuthbert et al., 2019). The CODD for each angulation (45º, 90º and 180º) was calculated using the formula: ([COD tests time – Ls time] / Ls time) * 100 (Freitas et al., 2021b); all time variables are provided in seconds.

### Unilateral Triple Hop Test (THTu)

The elastic-reactive force in a horizontal direction was evaluated through the horizontal triple jump test measured with a metric tape measure. The test started when the player stood with one leg supported just behind the starting line. After performing three consecutive maximum forward jumps with the same leg, the investigator measured the total distance jumped from the take- off line to the nearest point of the landing contact (i.e., back of the heels). Arm swinging was allowed and attempts were considered failed and repeated if 1) the test was not completed as previously indicated, 2) balance was lost during any part of the test, or 3) the final position could not be maintained on one leg for at least two seconds.

### Statistical Analyses

Means ± standard deviations (SDs) were used to describe variables. The assumption of normality was verified using the Shapiro-Wilk test. The relative and absolute reliability of the tests was evaluated by the ICC and the CV. A median cut-off score was established for the time execution of each Ls and COD tests, thus dividing participants according to their performance in each test. A high performance (HP) group included those participants with a performance above the 50^th^ percentile in each speed test, while a low performance (LP) group consisted of players with a performance below the 50^th^ percentile in each speed test. The Student's *t*-test for independent samples was used to assess the influence of gender and performance. Relationships between variables were determined using Pearson´s correlations. Finally, forward stepwise multiple regression analysis (MRA) was used to identify the effects of independent variables on performance in the different Ls and COD tests. These analyses were carried out separately for male and female players. The interpretation for Square R descriptors from MRA were < 0.04 ”trivial”, 0.04–0.25 ”small”, 0.25–0.64 ”moderate”, and > 0.64 ”strong effect” (Cohen, 1988). The Cohen’s *d* was calculated from the mean change divided by the SD of the data; thresholds for qualitative descriptors of Cohen’s *d* were set at < 0.20 ”trivial”, 0.20–0.50 ”small”, 0.50–0.80 ”moderate”, and > 0.80 ”large” (Cohen, 1988). Collinearity diagnostic tests were performed for each MRA and the thresholds for determining the presence of collinearity were set at ≤ 0.3 for tolerance and ≥ 3 for the variance inflation factor (VIF). The Breusch-Pagan test was employed to assess the presence of heteroscedasticity. Statistical significance was set at *p* < 0.05. All statistical analysis was performed using IBM SPSS Statistics for Macintosh, Version 25.0 (Armonk, NY: IBM Corp.).

## Results

The average performance of the different quantitative mobility, strength, Ls and COD tests differing according to gender is presented in Table 1. Male players showed a better performance for all the strength variables analysed (*p* < 0.001) as well as for velocity variables, with COD45º being the variable that showed the greatest difference between both genders (*p* < 0.001; ES = 2.047).

**Table 1:** Descriptive data (mean ± SD) of the considered performance variables differentiating between genders.

Variables	FEMALE (n = 27)		MALE (n = 23)		% difference	*p* value	ES Cohen’s *d*	CV (%)
	Mean	SD	Mean	SD				
WB-DF (º)	41.2	5.13	41.7	7.25	1.21	0.784	–0.079	1.32
YBT	0.61	0.05	0.60	0.06	1.67	0.877	0.047	1.81
CMJu (cm)	10.9	3.08	17.1	3.83	56.9	<0.001	–1.838	1.94
DJu (cm)	11.0	2.59	16.2	3.37	47.3	<0.001	–1.735	2.15
RSI	0.72	0.18	0.83	0.19	15.3	0.034	–0.618	2.07
THT (cm)	464.3	45.5	535.1	60.5	15.2	<0.001	–1.331	1.98
LS (s)	1.98	0.11	1.87	0.10	5.56	0.001	1.093	1.69
COD 45º (s)	2.12	0.10	1.90	0.11	10.4	<0.001	2.047	1.76
COD 90º (s)	2.40	0.14	2.31	0.14	3.75	0.022	0.695	1.85
COD 180º (s)	2.93	0.18	2.76	0.12	5.80	<0.001	1.138	1.62
CODD 45º (%)	7.08	3.60	2.00	4.74	71.8	<0.001	1.211	−
CODD 90º (%)	21.2	4.80	23.5	5.45	10.8	0.132	–0.448	−
CODD 180º (%)	47.9	6.30	47.9	6.77	0.00	0.990	0.004	−

WB-DF, Weight-Bearing Dorsiflexion Test; YBT, Y-Balance Test; CMJu, unilateral Countermovement Jump; DJu: unilateral Drop jump; RSI: Reactive Strength Index; THT, Triple Hop Test; LS, Linear Sprint; COD, Change of Direction; CODD, Change of Direction Deficit; º, Degrees; %, Percentage; cm, Centimeters; s, Seconds; SD, Standard Deviation; ES, Effect Size.

Comparison of performance variables differentiating between genders and according to performance in Ls and COD tests is shown in Table 2. For all speed tests performed, the fastest athletes were also the strongest both vertically (*p* < 0.021) and horizontally (*p <* 0.035). Vertical strength showed significant differences (*p* < 0.05) in the performance of all speed tests only among female players. However, significant differences were observed between horizontal strength and performance in all speed tests for both genders. Specifically, performance in Ls and COD90º tests showed a greater relationship with all force variables in both directions. For these variables, the fastest athletes showed a higher RSI (*p* < 0.05) in both genders. Finally, a higher CODD (*p* < 0.05) was also observed in the fastest players in Ls only in the male sample.

**Table 2:** Comparison of the different considered performance variables between low performance and high performance classified by each linear speed and change of direction test and differentiating between genders.

	LS		COD 45°		COD 90°		COD 180°	
	LP	HP	LP	HP	LP	HP	LP	HP
**WB-DF** (°)								
Female	39.1 ± 5.62	42.7 ± 4.65	38.8 ± 5.56	43.0 ± 4.47	40.1 ± 6.22	41.8 ± 4.50	40.9 ± 6.35	41.0 ± 4.48
Male	38.8 ± 5.72	44.6 ± 7.69	42.8 ± 8.18	40.8 ± 6.61	40.6 ± 8.10	42.8 ± 6.50	38.5 ± 6.00	44.3 ± 7.37
*p* gend	0.665		0.644		0.687		0.782	
*p* perf	0.011		0.556		0.323		0.125	
**YBT**								
Female	0.58 ± 0.06^a^	0.65 ± 0.05^a^	0.58 ± 0.05^a^	0.64 ± 0.06^a^	0.57 ± 0.05^a^	0.65 ± 0.05^a^	0.58 ± 0.06^a^	0.64 ± 0.04^a^
Male	0.60 ± 0.05	0.63 ± 0.08	0.61 ± 0.06	0.60 ± 0.07	0.62 ± 0.06	0.60 ± 0.07	0.61 ± 0.02	0.60 ± 0.09
*p* gend	0.487		0.621		0.652		0.458	
*p* perf	0.006		0.077		0.038		0.041	
**CMJu** (cm)								
Female	8.98 ± 3.45^1a^	12.8 ±1.51^2a^	9.35 ± 3.70^1^a	12.5 ± 1.82^2a^	8.72 ± 2.95^1a^	13.1 ± 1.76^2^a	9.36 ± 3.41^1a^	12.5 ± 2.33^2a^
Male	15.3 ± 3.04^1b^	20.5 ± 2.84^2b^	16.1 ± 2.82^1^	18.2 ± 4.72^2^	15.3 ± 2.30^1^	18.6 ± 4.39^2^	15.8 ± 4.26^1^	18.5 ± 2.91^2^
*p* gend	< 0.001		< 0.001		< 0.001		< 0.001	
*p* perf	< 0.001		0.021		< 0.001		0.009	
**DJu** (cm)								
Female	9.06 ± 1.94^1a^	13.0 ± 1.81^2a^	9.72 ± 2.80^1a^	12.4 ± 1.98^2a^	9.08 ± 1.83^1^a	13.0 ± 1.97^2a^	9.60 ± 2.53^1a^	12.5 ± 2.16^2a^
Male	14.9 ± 2.85^1^	17.3 ± 3.50^2^	14.8 ± 1.69^1^	17.5 ± 4.05^2^	14.7 ± 1.56^1b^	17.5 ± 4.04^2b^	15.2 ± 3.49^1^	17.1 ± 3.10^2^
*p* gend	< 0.001		< 0.001		< 0.001		< 0.001	
*p* perf	< 0.001		0.002		< 0.001		0.006	
**RSI**								
Female	0.63 ± 0.13^1a^	0.77 ± 0.18^a^	0.66 ± 0.21	0.74 ± 0.12	0.61 ± 0.11^1a^	0.80 ± 0.17^a^	0.68 ± 0.23	0.72 ± 0.08^2^
Male	0.78 ± 0.17^1^	0.87 ± 0.21	0.80 ± 0.18	0.86 ± 0.21	0.74 ± 0.13^1b^	0.92 ± 0.20^b^	0.81 ± 0.21	0.85 ± 0.18^2^
*p* gend	0.016		0.020		0.009		0.021	
*p* perf	0.028		0.226		< 0.001		0.461	
**THT** (cm)								
Female	434.6 ± 37.4^1a^	492.0±35.7^2a^	441.3±40.5^1a^	485.4±42.0^2a^	440.6±40.9^1a^	486.0±40.9^2a^	445.1±44.8^1a^	481.5±41.7^2a^
Male	504.9 ± 51.1^1b^	562.9±56.5^2b^	522.9±61.0^1^	546.3±60.5^2^	500.4±51.3^1b^	566.9±51.2^2b^	509.2±58.6^1b^	558.9±53.9^2b^
*p* gend	< 0.001		< 0.001		< 0.001		< 0.001	
*p* perf	< 0.001		0.031		< 0.001		0.005	
**CODD** (%)								
Female	23.9 ± 4.19	26.9 ± 3.72	7.36 ± 3.92	6.80 ± 3.40^2^	22.2 ± 5.23^1^	20.3 ± 4.35	50.4 ± 5.24	45.5 ± 6.55
Male	21.2 ± 4.61^b^	27.5 ± 2.54^b^	3.56 ± 5.69	0.57 ± 3.29^2^	26.6 ± 3.52^1b^	20.7 ± 5.50^b^	48.2 ± 8.76	47.7 ± 4.66
*p* gend	0.342		< 0.001		0.087		0.994	
*p* perf	< 0.001		0.150		0.008		0.171	

LP, Low Performance; HP, High Performance; LS, Linear Speed; COD, Change of Direction; gend, gender; perf, performance; WB-DF, Weight-Bearing Dorsiflexion Test; YBT, Y-Balance Test; CMJu, unilateral Countermovement Jump; DJu: unilateral Drop Jump; RSI: Reactive Strength Index; THT, Triple Hop Test; CODD, Change of Direction Deficit; º, Degrees; %, Percentage; cm, Centimeters; s, Seconds; SD, Standard Deviation. 1 Statistical significance at p < 0.05 in LP controlling for gender. 2 Statistical significance at p < 0.05 in HP controlling for gender. a Statistical significance at p < 0.05 in females controlling for performance. b Statistical significance at p < 0.05 in males controlling for performance.

Matrix correlation analysis is presented in Table 3. This analysis revealed that the strength variables evaluated had greater statistical significance in females compared to males. In females, the CMJu showed the highest significant correlations (r ≤ –0.684; *p* < 0.01) with all speed tests. In the male sample, the CMJu, the DJu and the THTu showed significant relationships (*p* < 0.05) with several speed tests. Quantitative movement tests only showed significant relationships with speed performance for female gender. The YBT showed high negative relationships with all speed tests (r ≤ –0.573; *p* < 0.01) and the WB-DT was only significantly related to COD45° (*p* < 0.01).

**Table 3:** Correlation coefficients between the movement and performance variables with the linear speed and change of direction performance according to gender.

Variables	LS		COD 45º		COD 90º		COD 180º	
	Male	Female	Male	Female	Male	Female	Male	Female
**WB-DF** (º)	–0.252	–0.270	–0.003	–0.533^††^	–0.152	–0.282	–0.217	–0.299
**YBT** (cm)	–0.177	–0.597^††^	–0.181	–0.573^††^	–0.015	–0.607^††^	0.018	–0.625^††^
**CMJu** (cm)	–0.678^††^	–0.730^††^	–0.401	–0.750^††^	–0.417	–0.792^††^	–0.538^†^	–0.684^††^
**DJu** (cm)	–0.513^†^	–0.829^††^	–0.488^†^	–0.666^††^	–0.455^†^	–0.701^††^	–0.511^†^	–0.642^††^
**RSI**	–0.403	–0.450^†^	–0.252	–0.493^††^	–0.379	–0.533^††^	–0.318	–0.307
**THT** (cm)	–0.607^††^	–0.663^††^	–0.397	–0.679^††^	–0.561^††^	–0.655^††^	–0.512^†^	–0.640^††^

LS, Linear Speed; COD, Change of Direction; WB-DF, Weight-Bearing Dorsiflexion Test; YBT, Y-Balance Test; CMJu, unilateral Countermovement Jump; DJu: unilateral Drop Jump; RSI: Reactive Strength Index; THT, unilateral Triple Hop Test. ^†^ Significant correlation (*p* < 0.05); ^††^ Significant correlation (*p* < 0.01)

Finally, Table 4 shows MRA, revealing the variance and properties of each speed test based on gender using independent variables. For females, the CMJu explained between 45% and 68% (*p* < 0.001) of the performance in speed tests, being the most influential variable. On the other hand, for male players, the THTu emerged as the most significant variable, explaining up to 61% (*p* < 0.001) of their performance in the different speed tests. Collinearity was not detected in MRA (tolerance ≥ 0.92, VIF ≤ 1.09 for females; tolerance ≥ 0.95, VIF ≤ 1.02 for males). Finally, no heteroscedasticity was detected in the regression model (*p* ≥ 0.145 for females; *p* ≥ 0.346 for males).

**Table 4:** Stepwise multiple regression analysis between performance variables assessed and linear-change of direction speed performance.

Dependent variable	Independent variables	Standardised coefficients (Beta)	Coefficients’ significance (*p*)	R^2^	R^2^ adjusted
Female					
LS	DJu	–0.829	<0.001	0.687	0.673
COD 45º	CMJu	–0.652	<0.001	0.675	0.644
	WB-DF	–0.348	0.014		
COD 90º	CMJu	–0.792	<0.001	0.628	0.611
COD 180º	CMJu	–0.684	<0.001	0.467	0.443
Male					
LS	THT	–0.796	<0.001	0.634	0.606
COD 45º	DJu	–0.488	0.018	0.238	0.201
COD 90º	THT	–0.585	0.022	0.342	0.292
COD 180º	THT	–0.770	0.001	0.593	0.561

R^2^, Coefficient of determination; LS, Linear Speed; COD, Change of Direction; WB-DF, Weight-Bearing Dorsiflexion Test; CMJu, unilateral Countermovement Jump; DJu: unilateral Drop Jump; THT, Triple Hop Test.

## Discussion

This study aimed to analyse the relationship among functional movement and strength variables with Ls and COD speed based on gender. The main findings were as follows: 1) greater performance in all jumps actions was associated with faster athletes in all speed tests. Horizontal jumps showed a stronger association with speed tests in males, while vertical jumps were more significant in females; 2) the CODD showed significant differences only in male players, suggesting that faster linear sprinters were less efficient in COD actions and had a higher CODD. In addition, the study also identified the main variables determining Ls and COD performance according to gender. In this sense, for males, 3) tests with a short SSC predominantly in a horizontal direction, like a THTu, were most significant for speed test performance, except for the COD45º. However, for females, 4) a long SSC in a vertical direction was the primary determinant of performance in all speed tests, with the WD-DF also significant for the COD45º.

As expected and consistent with prior literature (Freitas et al., 2021a; Mancha-Triguero et al., 2021; Mikolajec et al., 2017; Sekulic et al., 2013), males performed better than females in all strength and speed tests considered. These differences can be attributed to variations in body dimensions and muscle architecture (e.g., disparities in tendon length, angle of pennation and fascicle properties) between genders (Laffaye et al., 2014). In contrast, while previous studies observed gender differences in dynamic balance (Sekulic et al., 2013), no gender differences in quantitative movement tests were observed in the present study.

For the female sample, all strength variables in different directions significantly differed between performance levels for both Ls and COD speed tests, aligning with previous research (Spiteri et al., 2013, 2015). Further analysis using MRA revealed that the CMJu, involving vertical forces through a long SSC (> 250 ms) (Turner and Jeffreys, 2010), was the most significant variable determining speed performance in female athletes. While previous studies emphasized the importance of developing all strength components for female basketball players (Spiteri et al., 2014, 2015), our findings suggest that to improve COD performance in females, maximum strength exercises with vertical directions should be of the prime focus. This is at odds with previous research (Falch et al., 2020) that favoured exercises with a short SSC as more specific to the COD. The discrepancy could be due to a longer SSC being more relevant to COD actions in athletes with lower relative strength, as observed in the female sample compared to the male one in this study. In relation to this, it was suggested that there might be a strength threshold above which athletes benefit more from short SSC exercises (Falch et al., 2021), but below this threshold, the focus should be on maximal strength and long SSC exercises. Furthermore, it was initially hypothesised that speed tests would emphasise horizontal directions force, but our results showed just the contrary. This finding could be explained by the fact that vertically oriented exercises have previously been demonstrated to be more effective in improving Ls and COD performance (Zweifel, 2017); and this effectiveness was higher in less experienced and weaker athletes, such as female players in our sample.

In the male sample, significant differences were observed between performance in all speed tests and horizontal strength variables with a short SSC (< 250 ms) (Turner and Jeffreys, 2010). Specifically, the THTu had the greatest impact on speed performance in MRA. This could be because the analysed Ls and COD tests required a greater application of force in a horizontal direction (Baena-Raya et al., 2020; McBurnie and Dos’Santos, 2022; Robles-Ruiz et al., 2021). Previous interventions with male basketball players have shown that horizontal plyometric training leads to greater improvements in COD performance compared to vertical training (Aztarain-Cardiel et al., 2022; Gonzalo-Skok et al., 2019). Moreover, male players exhibited higher levels of strength, enabling them to take better advantage of a shorter SSC compared to female players. This suggests that males benefit more from a short SSC, as in previous research they have been shown to have a 1.5 times greater rate of force development than females (Laffaye et al., 2014). Finally, among the quantitative movement tests assessed, the YBT showed significant differences for all speed test performance variables, but only in the female sample. In contrast, no relationship was found between performance in speed and quantitative movement tests for male players, which differs from previous research that observed correlations between the YBT and speed tests in male basketball players (Gonzalo-Skok et al., 2015). This lack of consensus may be explained by the use of different speed tests, as each test has its own biomechanical and physical demands (Dos’Santos et al., 2018).

Although Ls and COD speed are considered to be two different capacities (Young et al., 2015), it is now accepted in the literature that one affects another, mainly regarding the lower cutting angle (Young et al., 2015). Previous research (Nimphius et al., 2016, 2018) states that the COD used in the literature may not isolate the COD ability, leading to observed relationships between Ls and COD speed possibly due to inadequate test variables, with over 70% of the total test time being devoted to Ls. Caution is advised when comparing these two variables, and using them as dependent variables when comparing them with other performance variables is recommended. To address this limitation, the CODD concept was proposed (Freitas et al., 2021b). Our results showed significant differences in the CODD for males, indicating less efficiency in COD actions despite their higher Ls. This relationship was not observed among females, possibly due to males having greater Ls development and higher body mass, which are important factors influencing the magnitude of the CODD (Freitas et al., 2022) and the braking phase of COD (Dos’Santos et al., 2018). While there are no basketball-specific studies comparing the CODD between genders, similar findings were obtained in both genders in previous research with rugby seven players (Freitas et al., 2021a).

This study is the first to comprehensively analyse the relationship between different movement and strength variables, previously linked to speed tests, and their association with various Ls and COD tests in both male and female players. This approach enables a comprehensive understanding of the performance variables and their relationship with speed tests with diverse angulations according to gender. However, despite its value, this research is not without limitations. Firstly, the cross-sectional design used precludes determining the causality of the results. In addition, we must take into account the characteristics of the sample before generalizing the results to other competitive levels or sports. Therefore, further prospective and experimental research is recommended to consistently determine gender differences. Therefore, since COD actions are complex and influenced by various factors, future research should explore different pre-COD running distances to modify finishing speed. Considering the vast array of performance variables influencing COD actions, this study had to carefully select the most valid and consistent ones for analysis.

## Conclusions

In summary, it is worth emphasizing that the influence of gender must be taken into account when optimizing Ls and COD speed. In this sense, it is recommended to focus on a short SSC and horizontal force direction exercises in men, and a long SSC including maximal strength with vertical force direction exercises in women. As MRA showed, the CMJu involving vertical force direction and a long SSC was the most determinant variable in speed performance for female athletes, explaining the variance of the applied tests between 44.3% and 67.3%. In contrast, the THTu with a horizontal force direction and a short SSC was the most determinant variable in speed performance of male athletes, explaining between 20.1% and 60.6% of the change in their variance. For further individualization of training, it is also recommended to take into account individual strength levels of each male and female athlete. Moreover, coaches should start using the CODD as an isolated measure of COD actions. It was established that male athletes who presented higher linear speed had a greater deficit in multidirectional actions. Thus, we recommend greater focus on eccentric actions, deceleration and technique drills in those male players who have better performance in Ls in order to decrease the CODD (Freitas et al., 2021b; Lazić et al., 2022) and, therefore, improve COD performance.
